# Editorial: A Feasible Approach for Natural Products to Treatment of Diseases

**DOI:** 10.3390/molecules28093791

**Published:** 2023-04-28

**Authors:** Soo Liang Ooi, Sok Cheon Pak

**Affiliations:** School of Dentistry and Medical Sciences, Charles Sturt University, Bathurst, NSW 2795, Australia; sooi@csu.edu.au

The potential of natural products from both plant and animal sources to treat diseases remains enormous, as our understating forms just the tip of the iceberg. There are also other issues to address regarding extraction, isolation, and standardization of any naturally derived compounds and their effective mode of therapeutic delivery. Hence, much research is needed before any natural products can be feasibly applied in treatments. The aim of this Special Issue was to first bring together academics and researchers whose work focuses on natural products to explore and debate different therapeutic perspectives, methodological approaches, and analytical findings. The second aim was to explore key developments and highlight new areas of research and ideas emerging on natural products. Finally, the third aim was to highlight and debate the potential translation of research from the bench to the bedside, which may directly benefit humans.

The opening article by Zhang et al. [1] explores a novel class of antifungal antibiotics from *Icacina trichantha* Oliv., a potential plant source of various bioactive endophytes. The authors document the endophytic fungus *Penicillium oxalicum* derived from the *Icacina* species and its related bioactivity. A novel macrolactam named oxalactam A is detailed, and dipeptides and alkaloids were isolated from this fungus. The article describes the isolation, structural elucidation, and anti-fungal activities of these isolates. These findings indicate an alternative natural source of new fungicides. 

Somwong et al. [2] next explore the underlying mechanisms of the fresh stem bark extract of *Holoptelea integrifolia* (Roxb.) Planch. in the context of treating human cutaneous diseases. They used thin layer and gas chromatography to screen phytochemicals from this sample. Two bioactive compounds, friedelin and lupeol, were identified, and their activity in wound healing was investigated in keratinocytes. Both compounds exhibited wound healing activity by increasing keratinocyte migration and matrix metalloproteinase-9 production. In addition, gene expressions for wound healing and pro-inflammation were increased and reduced, respectively, after treatment with *n*-hexane extracts of *H. integrifolia* and its bioactive compounds. Thus, the wound healing and anti-inflammation properties are mediated by regulating the gene expression involved in skin re-epithelialization. 

As major active compounds from *Angelica sinensis* and *Astragalus membranaceus*, *Angelica sinensis* polysaccharide (ASP) and *Astragalus membranaceus* polysaccharide (AMP) are known to exert anti-fibrosis and hepatoprotective effects. Wen et al. [3] studied their roles and related mechanisms in liver regeneration. The study demonstrated that ASP and AMP promoted hepatocyte proliferation at various concentrations in vitro while inducing hepatoprotection of liver injuries, demonstrating an enhanced liver/body weight ratio and reduced serum transaminase and total bilirubin levels after partial hepatectomy in mice. Further analyses confirmed the involvement of the JAK2/STAT3/HK2 pathway in ASP- and AMP-accelerated liver regeneration. These results indicate ASP and AMP as potential hepatoprotective agents. 

In another combined in vivo and in vitro experiment, Moon et al. [4] showed that walnuts (*Juglans regia*) could be a potential functional food to improve diabetic cognitive deficits and neuronal impairments. The study found evidence that walnuts inhibit reactive oxygen species production in high-glucose-induced neuronal and hippocampal cells and ameliorate behavioral and memory dysfunction in mice with cognitive impairment induced by a high fat diet. Further amelioration of cerebral damage with walnut treatment was observed via protein expression of the JNK signaling and apoptosis pathways. Hence, walnuts could protect against cerebral disorders, insulin resistance, oxidative stress, and inflammation. 

*Molineria recurvata* (MR) is used to manage diabetes mellitus. Dey et al. [5] investigated the protective effects of MR extracts versus nephropathy in streptozotocin-induced diabetic rats. The MR extracts showed anti-fibrotic properties, demonstrated by downregulated expressions of fibronection, collagen, and α-smooth muscle actin. Increased oxidative stress caused by induced hyperglycemia was improved after administration of MR extracts, with the marked restoration of antioxidant enzymes. Diabetic-induced renal injuries with accompanying inflammation were also ameliorated by MR extracts, with increased levels of SIRT1 and SIRT3 and a reduced claudin-1 level in the kidneys. Thus, MR could promote renal repair by repressing inflammation and oxidative stress.

Idriss et al. [6] investigated inhibitors from *Saussurea costus* against the main protease, as it is an ideal target for COVID-19 treatment. The authors found that eight of the active inhibitors were carbohydrates, five were fatty acids, three were terpenoids, two were carboxylic acids, one was a tannin, one was a phenolic compound, and one was a sterol. In addition, the *Saussurea costus* aqueous extract had no virucidal effect and inhibited the virus after cell entry. As such, the findings justify using this plant, especially in rural communities, for preventing and treating COVID-19.

As a flagellated parasitic protozoan, *Trypanosoma brucei* causes African trypanosomiasis, a neglected tropical disease. According to Chaudhuri et al. [7], two natural antimetabolites, cholesta-5,7,22,24-tetraenol (CHT) and ergosta-5,7,22,24(28)-tetraenol (ERGT), possess antiprotozoal properties as a trypanocidal agent against *T. brucei*. In addition, CHT/ERGT protected mice infected with *T. brucei* by increasing their survival time. Hence, CHT and ERGT are promising candidates for antitrypanosomal drugs. 

When food products containing arginine, glucose, or maltose undergo heat processing, arginyl-fructose (AF) and arginyl-fructosyl-glucose (AFG) are formed. It is known that AF and AFG can modulate the glucose uptake from dietary carbohydrates by exhibiting an inhibitory effect on α-glucosidases. Lee et al. [8] investigated AFs anti-hyperglycemic effect in barley using AF-enriched barley extract (BEE) in mice. Mice who were fed a high fat diet and treated with BEE showed a suppressed body weight gain and increased serum adiponectin levels. The accumulation of intracellular lipids and the expressions of adipogenic genes in the preadipocytes were significantly decreased after treatment with BEE. Thus, BEE could be used as a weight management strategy by inhibiting adipogenesis and increasing adiponectin levels. 

Hordyjewska et al. [9] critically examined whether electric cell-substrate impedance sensing (ECIS) could be a valuable tool for live monitoring of changes in the morphology and physiology of cancer cells. Betulin and betulinic acid are naturally occurring pentacyclic lupane-type triterpenoids, possessing a broad spectrum of biological activities, including antitumor activities. The ECIS results confirmed the great potential effects of betulin and betulinic acid’s antitumor properties on lung and breast cancer cell lines. Moreover, both substances showed a negligible toxic effect on healthy epithelial cells. The ECIS method is an appropriate alternative to the currently used assay for testing the in vitro anticancer activity of compounds.

Lemieszek et al. [10] bring our attention to the significance of young barley on the human body in terms of colon cancer prevention. The water extracts of young green barley (*Hordeum vulgare*) were evaluated for their immunoenhancement properties. Polysaccharide-rich young green barley extracts may have immunomodulatory properties associated with enhancing the natural killer cells’ ability to recognize and eliminate human colon cancer cells without any side effects on normal colon epithelial cells. The findings indicate the beneficial effect of the consumption of young barley regarding colon cancer. 

This Special Issue includes three review articles. Yao and Liu [11] offer a comprehensive review of terpenoids, focusing on non-alcoholic fatty liver disease (NAFLD). Despite a lack of clinical research, the authors found that terpenoids could have a therapeutic role in NAFLD by regulating lipid metabolism, insulin resistance, oxidative stress, and inflammation. 

Song et al. [12] introduce Citri Reticulatae Pericarpium (CRP), derived from the ripe peel of the Rutaceae plant *Citrus reticulate Blanco* and its cultivars. It is often used to treat diseases with coughs, expectoration, nausea, and vomiting as the main symptoms. The authors review the pharmacology of CRP and the mechanism of flavonoids as the key components of CRP against cancers with a high diagnosis rate. Although CRP does not prevent certain aspects of cancer, it reverses or suppresses the development of cancer through various pathways. 

Another review article by Phillips et al. [13] explores the current research on medicinal mushrooms in the context of COVID-19. The authors identify the key properties claimed to confer health benefits. Also considered are the barriers or limitations that may impact the general recommendations of medicinal mushrooms as a therapy. Notably, the authors include mushrooms commonly available for culinary use and obtainable as a dietary supplement for medicinal purposes. 

In the last few decades, there has been a wealth of research into natural products. This Special Issue has brought together active researchers who have explored a diverse range of products and provided rich insights into their underlying mechanisms. We believe that further preclinical and clinical research is still required to uncover the active constituents, mechanisms of action, pharmacokinetics, safety, and toxicity of natural products for therapeutic use. The diverse and critical perspectives within this Special Issue provide a springboard to enable the continual development and enhancement of natural products in therapeutic applications.
